# ([2.2.2]Cryptand-κ^6^ *O*)potassium (η^4^-cyclo­octa­diene)bis­(η^2^-pyrene)cobaltate(1−) pentane hemisolvate 

**DOI:** 10.1107/S1600536812029078

**Published:** 2012-06-30

**Authors:** William W. Brennessel, John E. Ellis

**Affiliations:** a Department of Chemistry, 207 Pleasant Street SE, University of Minnesota, Minneapolis, MN 55455, USA

## Abstract

The cation, anion, and solvent in the title compound, [K(C_18_H_36_N_2_O_6_)][Co(C_8_H_12_)(C_16_H_10_)_2_]·0.5C_5_H_12_, are well separated. The pentane solvent mol­ecules are found in channels along [100] and were modeled as disordered over crystallographic inversion centers. Using the mid-points of the coordinated olefins, the angle between the C_py_/C_py_–Co–C_py_/C_py_ and the C_cod_/C_cod_–Co–C_cod_/C_cod_ planes (py is pyrene and cod is cyclo­octa­diene) is 67.6 (2)°. Thus, the overall geometry of the coordination sphere around cobalt is best described as distorted tetra­hedral.

## Related literature

For the synthesis of the precursor mol­ecule, see: Brennessel *et al.* (2006 ▶). For cobalt anions with non-conjugated olefin ligands, see: Jonas (1981 ▶, 1984 ▶, 1985 ▶); Jonas *et al.* (1976 ▶); Jonas & Krüger (1980 ▶). For the structure of free pyrene, see: Frampton *et al.* (2000 ▶). For a description of the Cambridge Structural Database, see: Allen (2002 ▶).
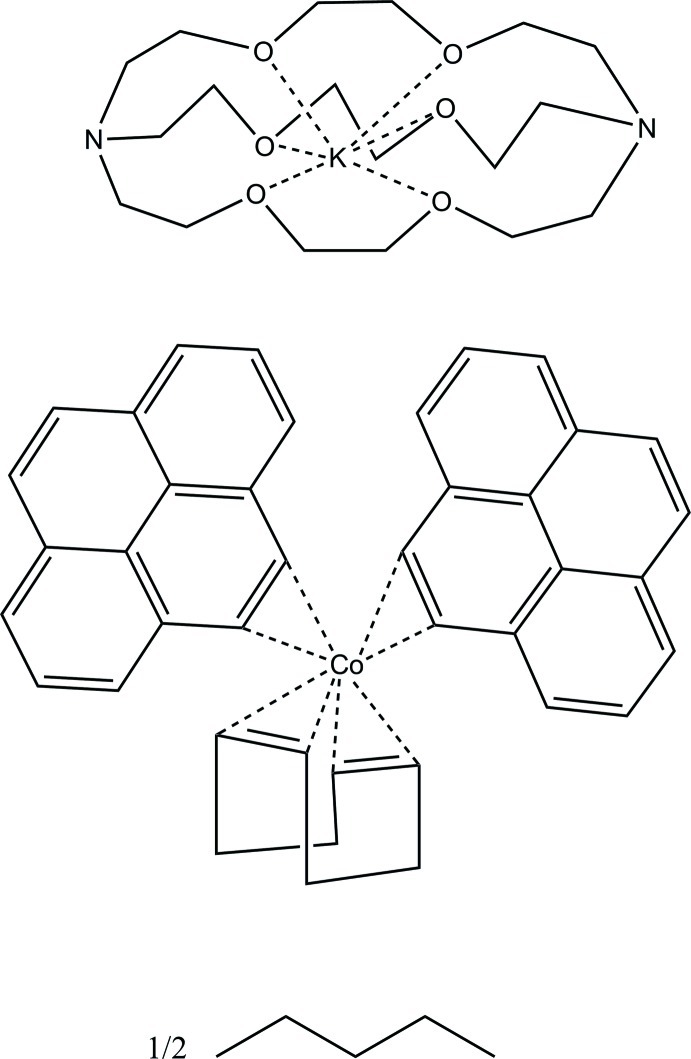


## Experimental

### 

#### Crystal data

[K(C_18_H_36_N_2_O_6_)][Co(C_8_H_12_)(C_16_H_10_)_2_]·0.5C_5_H_12_
*M* *_r_* = 1023.25
Triclinic, 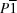

*a* = 12.1007 (9) Å
*b* = 12.9869 (10) Å
*c* = 18.7501 (14) Å
α = 72.064 (1)°
β = 81.595 (1)°
γ = 68.558 (1)°
*V* = 2607.5 (3) Å^3^
*Z* = 2
Mo *K*α radiation
μ = 0.46 mm^−1^
*T* = 173 K
0.40 × 0.24 × 0.08 mm

#### Data collection

Bruker SMART CCD Platform diffractometer
Absorption correction: multi-scan (*SADABS*; Sheldrick, 2008 ▶) *T*_min_ = 0.837, *T*_max_ = 0.964
29850 measured reflections
11375 independent reflections
7919 reflections with *I* > 2σ(*I*)
*R*_int_ = 0.037

#### Refinement

*R*[*F* ^2^ > 2σ(*F* ^2^)] = 0.042
*wR*(*F* ^2^) = 0.108
*S* = 1.01
11375 reflections
678 parameters
5 restraints
H atoms treated by a mixture of independent and constrained refinement
Δρ_max_ = 0.74 e Å^−3^
Δρ_min_ = −0.48 e Å^−3^

### 

Data collection: *SMART* (Bruker, 2003 ▶); cell refinement: *SAINT* (Bruker, 2003 ▶); data reduction: *SAINT*; program(s) used to solve structure: *SIR97* (Altomare *et al.*, 1999 ▶); program(s) used to refine structure: *SHELXL97* (Sheldrick, 2008 ▶); molecular graphics: *SHELXTL* (Sheldrick, 2008 ▶); software used to prepare material for publication: *SHELXTL*.

## Supplementary Material

Crystal structure: contains datablock(s) I, global. DOI: 10.1107/S1600536812029078/im2388sup1.cif

Structure factors: contains datablock(s) I. DOI: 10.1107/S1600536812029078/im2388Isup2.hkl

Additional supplementary materials:  crystallographic information; 3D view; checkCIF report

## Figures and Tables

**Fig. 1.**
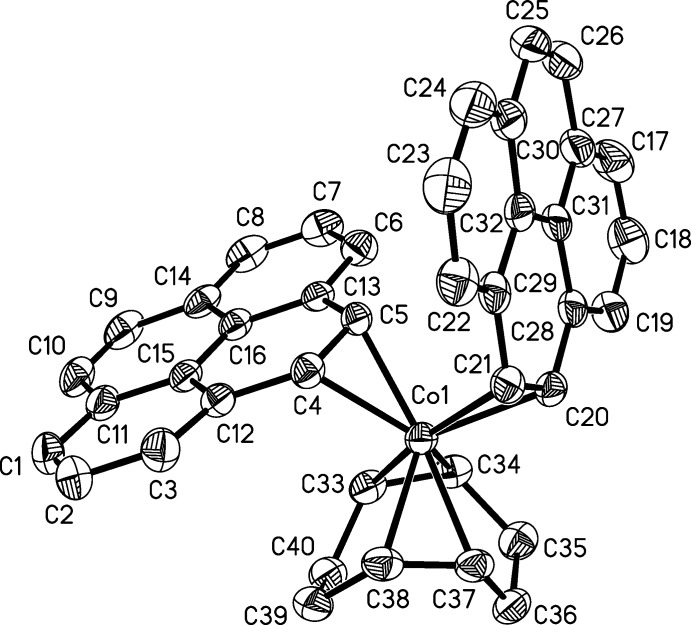
Molecular structure of the anion showing displacement ellipsoids at the 50% probability level.

## supplementary crystallographic information

### Comment

Klaus Jonas had shown that cobalt anions could be ligated solely by olefinic moieties, whether by ethylene itself or by isolobal analogs like 1,5-cyclooctadiene (Jonas *et al.*, 1976; Jonas & Krüger, 1980; Jonas, 1981, 1984, 1985). This led to the possibility that η^2^-coordinated pyrene ligands could offer a similar stabilization of the metal center. Pyrene is an interesting molecule in that when coordinated η^2^ through two side carbon atoms (C4/C5 and C20/C21 in figure), it can be behave as a coordinated ethylene ligand with an *exo*-phenanthrene unit. Thus pyrene could offer the ethylene-type ligation, with the possible bonuses of resonance stabilization energy in the *exo*-phenanthrene portion and additional electron withdrawing capabilities.

The structure is unique. Of the ten reported transition metal structures to date containing a pyrene or substituted (not by additional fused rings) pyrene ligand (Allen, 2002), only the title compound is mononuclear and has a pyrene-to-transition metal ratio of greater than one. The coordinated ethylene moieties of the pyrene ligands have C–C bond distances of 1.420 (3) and 1.425 (3) Å, which are much longer than the respective distances in free pyrene (average 1.341 Å) (Frampton *et al.*, 2000), and provide evidence of significant back-bonding.

### Experimental

Room temperature tetrahydrofuran (THF) was added to a flask containing excess cryptand[2.2.2], excess pyrene, and bright magenta and highly air-sensitive [K(THF)*_x_*][Co(η^4^-1,5-cyclooctadiene)(η^4^-C_10_H_8_)] (Brennessel *et al.*, 2006). The reaction mixture was swirled briefly, layered with pentane, and stored at 0 °C for two days. During that time, purple-black plates formed that were suitable for single-crystal X-ray diffraction.

### Refinement

Hydrogen atoms on metal-coordinated carbon atoms were found from the difference Fourier map, and their positional and isotropic displacement parameters were refined independently from those of their respective bonded carbon atoms. All other hydrogen atoms were placed geometrically, and refined relative to their respective bonded carbon atoms: *U*_iso_[H]= 1.5**U*_eq_[C] for methyl and *U*_iso_[H] = 1.2**U*_eq_[C] for methylene and aromatic hydrogen atoms.

The cocrystallized pentane solvent molecule is modeled as disordered over a crystallographic inversion center (50:50). Corresponding bond lengths and angles in both directions along the pentane molecule were restrained to be similar. Anisotropic displacement parameters for spatially close symmetry-related atoms were constrained to be equivalent. Bond lengths C59–C60 and C60–C61 were restrained toward ideal distances (1.52 Å).

### Crystal data

**Table:**

[K(C_18_H_36_N_2_O_6_)][Co(C_8_H_12_)(C_16_H_10_)_2_]·0.5C_5_H_12_	*Z* = 2
*M**_r_* = 1023.25	*F*(000) = 1090
Triclinic, *P*1	*D*_x_ = 1.303 Mg m^−^^3^
Hall symbol: -P 1	Mo *K*α radiation, λ = 0.71073 Å
*a* = 12.1007 (9) Å	Cell parameters from 3044 reflections
*b* = 12.9869 (10) Å	θ = 2.5–26.3°
*c* = 18.7501 (14) Å	µ = 0.46 mm^−^^1^
α = 72.064 (1)°	*T* = 173 K
β = 81.595 (1)°	Plate, purple-black
γ = 68.558 (1)°	0.40 × 0.24 × 0.08 mm
*V* = 2607.5 (3) Å^3^	

### Data collection

**Table:**

Bruker SMART CCD Platform diffractometer	11375 independent reflections
Radiation source: normal-focus sealed tube	7919 reflections with *I* > 2σ(*I*)
Graphite monochromator	*R*_int_ = 0.037
area detector, ω scans per φ	θ_max_ = 27.1°, θ_min_ = 1.8°
Absorption correction: multi-scan (*SADABS*; Sheldrick, 2008)	*h* = −15→15
*T*_min_ = 0.837, *T*_max_ = 0.964	*k* = −16→16
29850 measured reflections	*l* = −23→23

### Refinement

**Table:**

Refinement on *F*^2^	Primary atom site location: structure-invariant direct methods
Least-squares matrix: full	Secondary atom site location: difference Fourier map
*R*[*F*^2^ > 2σ(*F*^2^)] = 0.042	Hydrogen site location: inferred from neighbouring sites
*wR*(*F*^2^) = 0.108	H atoms treated by a mixture of independent and constrained refinement
*S* = 1.01	*w* = 1/[σ^2^(*F*_o_^2^) + (0.0421*P*)^2^ + 1.344*P*] where *P* = (*F*_o_^2^ + 2*F*_c_^2^)/3
11375 reflections	(Δ/σ)_max_ = 0.001
678 parameters	Δρ_max_ = 0.74 e Å^−^^3^
5 restraints	Δρ_min_ = −0.48 e Å^−^^3^

### Special details

**Table:**

Geometry. All e.s.d.'s (except the e.s.d. in the dihedral angle between two l.s. planes) are estimated using the full covariance matrix. The cell e.s.d.'s are taken into account individually in the estimation of e.s.d.'s in distances, angles and torsion angles; correlations between e.s.d.'s in cell parameters are only used when they are defined by crystal symmetry. An approximate (isotropic) treatment of cell e.s.d.'s is used for estimating e.s.d.'s involving l.s. planes.
Refinement. Refinement of *F*^2^ against ALL reflections. The weighted *R*-factor *wR* and goodness of fit *S* are based on *F*^2^, conventional *R*-factors *R* are based on *F*, with *F* set to zero for negative *F*^2^. The threshold expression of *F*^2^ > σ(*F*^2^) is used only for calculating *R*-factors(gt) *etc*. and is not relevant to the choice of reflections for refinement. *R*-factors based on *F*^2^ are statistically about twice as large as those based on *F*, and *R*- factors based on ALL data will be even larger.

### Fractional atomic coordinates and isotropic or equivalent isotropic displacement parameters (Å^2^)

**Table:**

	*x*	*y*	*z*	*U*_iso_*/*U*_eq_	Occ. (<1)
Co1	0.33875 (3)	0.99538 (2)	0.695903 (16)	0.02569 (9)	
C1	0.6218 (2)	0.5743 (2)	0.63059 (16)	0.0444 (6)	
H1	0.6440	0.5021	0.6202	0.053*	
C2	0.6095 (2)	0.6726 (2)	0.57259 (15)	0.0451 (6)	
H2	0.6238	0.6679	0.5223	0.054*	
C3	0.5761 (2)	0.7792 (2)	0.58711 (14)	0.0386 (6)	
H3	0.5707	0.8459	0.5464	0.046*	
C4	0.51596 (19)	0.90131 (18)	0.67510 (13)	0.0273 (5)	
H4	0.544 (2)	0.958 (2)	0.6390 (13)	0.032 (6)*	
C5	0.49812 (19)	0.90716 (18)	0.75050 (12)	0.0264 (5)	
H5	0.5152 (18)	0.9648 (19)	0.7633 (12)	0.026 (6)*	
C6	0.5048 (2)	0.8047 (2)	0.88655 (13)	0.0362 (5)	
H6	0.4812	0.8765	0.8977	0.043*	
C7	0.5272 (2)	0.7032 (2)	0.94513 (14)	0.0411 (6)	
H7	0.5192	0.7066	0.9956	0.049*	
C8	0.5606 (2)	0.5986 (2)	0.93018 (14)	0.0399 (6)	
H8	0.5757	0.5302	0.9705	0.048*	
C9	0.6059 (2)	0.48408 (19)	0.83857 (16)	0.0409 (6)	
H9A	0.6190	0.4150	0.8782	0.049*	
C10	0.6191 (2)	0.4786 (2)	0.76748 (16)	0.0413 (6)	
H10A	0.6406	0.4057	0.7581	0.050*	
C11	0.6017 (2)	0.57987 (19)	0.70492 (15)	0.0354 (5)	
C12	0.55070 (19)	0.79043 (19)	0.65970 (13)	0.0296 (5)	
C13	0.51646 (18)	0.80227 (18)	0.81235 (12)	0.0268 (5)	
C14	0.57259 (19)	0.59134 (19)	0.85637 (14)	0.0336 (5)	
C15	0.56647 (18)	0.68839 (18)	0.72026 (13)	0.0289 (5)	
C16	0.55072 (18)	0.69424 (18)	0.79645 (13)	0.0281 (5)	
C17	0.3968 (3)	1.1287 (2)	0.94023 (15)	0.0476 (7)	
H17	0.4196	1.1225	0.9882	0.057*	
C18	0.2855 (3)	1.1296 (2)	0.93170 (15)	0.0484 (7)	
H18	0.2313	1.1257	0.9737	0.058*	
C19	0.2512 (2)	1.1360 (2)	0.86258 (14)	0.0395 (6)	
H19	0.1737	1.1367	0.8582	0.047*	
C20	0.2928 (2)	1.14793 (18)	0.72689 (13)	0.0289 (5)	
H20	0.209 (2)	1.1841 (18)	0.7192 (12)	0.027 (6)*	
C21	0.3748 (2)	1.15244 (18)	0.66417 (13)	0.0281 (5)	
H21	0.3493 (19)	1.1879 (19)	0.6153 (13)	0.028 (6)*	
C22	0.5656 (2)	1.17953 (19)	0.60968 (14)	0.0368 (6)	
H22	0.5452	1.1823	0.5619	0.044*	
C23	0.6723 (2)	1.1930 (2)	0.61679 (16)	0.0469 (7)	
H23	0.7227	1.2065	0.5737	0.056*	
C24	0.7055 (2)	1.1870 (2)	0.68517 (17)	0.0467 (7)	
H24	0.7789	1.1956	0.6892	0.056*	
C25	0.6641 (2)	1.1577 (2)	0.82256 (17)	0.0458 (7)	
H25	0.7388	1.1621	0.8282	0.055*	
C26	0.5916 (2)	1.1419 (2)	0.88334 (16)	0.0467 (7)	
H26	0.6171	1.1338	0.9310	0.056*	
C27	0.4768 (2)	1.1370 (2)	0.87815 (14)	0.0385 (6)	
C28	0.3279 (2)	1.14161 (18)	0.79916 (13)	0.0297 (5)	
C29	0.4885 (2)	1.16202 (17)	0.67139 (13)	0.0294 (5)	
C30	0.6317 (2)	1.16822 (19)	0.74938 (15)	0.0378 (6)	
C31	0.4425 (2)	1.14376 (18)	0.80709 (13)	0.0307 (5)	
C32	0.5217 (2)	1.15711 (17)	0.74237 (13)	0.0303 (5)	
C33	0.2752 (2)	0.85817 (19)	0.74074 (13)	0.0321 (5)	
H33	0.3383 (19)	0.7945 (19)	0.7704 (12)	0.025 (6)*	
C34	0.2124 (2)	0.9475 (2)	0.77208 (14)	0.0320 (5)	
H34	0.232 (2)	0.9431 (19)	0.8179 (13)	0.032 (6)*	
C35	0.0879 (2)	1.0284 (2)	0.74944 (14)	0.0383 (6)	
H35A	0.0660	1.0939	0.7713	0.046*	
H35B	0.0309	0.9870	0.7700	0.046*	
C36	0.0790 (2)	1.0741 (2)	0.66415 (14)	0.0381 (6)	
H36A	0.0534	1.0230	0.6456	0.046*	
H36B	0.0175	1.1516	0.6515	0.046*	
C37	0.1960 (2)	1.0814 (2)	0.62439 (13)	0.0321 (5)	
H37	0.1962 (19)	1.158 (2)	0.6021 (12)	0.031 (6)*	
C38	0.2813 (2)	0.9961 (2)	0.59624 (13)	0.0333 (5)	
H38	0.332 (2)	1.019 (2)	0.5558 (14)	0.037 (7)*	
C39	0.2674 (2)	0.8827 (2)	0.60230 (14)	0.0390 (6)	
H39A	0.3438	0.8298	0.5877	0.047*	
H39B	0.2073	0.8956	0.5669	0.047*	
C40	0.2291 (2)	0.8262 (2)	0.68208 (14)	0.0380 (6)	
H40A	0.1413	0.8513	0.6867	0.046*	
H40B	0.2600	0.7415	0.6914	0.046*	
K1	0.04995 (4)	0.61799 (4)	0.72291 (3)	0.03135 (12)	
N1	−0.0702 (2)	0.6880 (2)	0.86364 (12)	0.0449 (5)	
N2	0.1725 (2)	0.54589 (18)	0.58168 (12)	0.0440 (5)	
O1	−0.11276 (14)	0.84204 (14)	0.70849 (10)	0.0443 (4)	
O2	−0.01292 (16)	0.76423 (14)	0.58045 (10)	0.0450 (4)	
O3	−0.10080 (14)	0.50457 (14)	0.81502 (9)	0.0385 (4)	
O4	0.03226 (14)	0.42536 (14)	0.69175 (9)	0.0396 (4)	
O5	0.18022 (16)	0.56685 (16)	0.84697 (10)	0.0502 (5)	
O6	0.29744 (15)	0.51687 (14)	0.71414 (11)	0.0453 (4)	
C41	−0.1290 (3)	0.8133 (3)	0.84052 (19)	0.0658 (10)	
H41A	−0.0684	0.8505	0.8332	0.079*	
H41B	−0.1850	0.8366	0.8814	0.079*	
C42	−0.1957 (3)	0.8566 (2)	0.7698 (2)	0.0637 (9)	
H42A	−0.2497	0.8131	0.7737	0.076*	
H42B	−0.2442	0.9390	0.7618	0.076*	
C43	−0.1721 (2)	0.8815 (2)	0.63935 (19)	0.0577 (8)	
H43A	−0.2295	0.9605	0.6336	0.069*	
H43B	−0.2165	0.8307	0.6395	0.069*	
C44	−0.0833 (3)	0.8804 (2)	0.57631 (16)	0.0519 (7)	
H44A	−0.1237	0.9176	0.5280	0.062*	
H44B	−0.0324	0.9236	0.5795	0.062*	
C45	0.0782 (3)	0.7543 (2)	0.52382 (15)	0.0531 (7)	
H45A	0.1458	0.7686	0.5379	0.064*	
H45B	0.0482	0.8117	0.4759	0.064*	
C46	0.1173 (3)	0.6363 (3)	0.51506 (15)	0.0565 (8)	
H46A	0.0477	0.6223	0.5035	0.068*	
H46B	0.1749	0.6313	0.4719	0.068*	
C47	−0.1578 (2)	0.6298 (2)	0.89382 (15)	0.0468 (7)	
H47A	−0.2306	0.6720	0.8646	0.056*	
H47B	−0.1794	0.6319	0.9465	0.056*	
C48	−0.1122 (2)	0.5073 (2)	0.89102 (14)	0.0442 (6)	
H48A	−0.0340	0.4667	0.9144	0.053*	
H48B	−0.1678	0.4677	0.9193	0.053*	
C49	−0.0697 (2)	0.3906 (2)	0.80932 (16)	0.0465 (7)	
H49A	−0.1287	0.3557	0.8380	0.056*	
H49B	0.0092	0.3429	0.8306	0.056*	
C50	−0.0666 (2)	0.3946 (2)	0.72899 (16)	0.0471 (7)	
H50A	−0.0595	0.3183	0.7247	0.056*	
H50B	−0.1409	0.4518	0.7056	0.056*	
C51	0.0398 (2)	0.4303 (2)	0.61475 (15)	0.0447 (6)	
H51A	−0.0247	0.4983	0.5879	0.054*	
H51B	0.0312	0.3604	0.6089	0.054*	
C52	0.1589 (2)	0.4384 (2)	0.58261 (16)	0.0469 (7)	
H52A	0.2222	0.3731	0.6126	0.056*	
H52B	0.1693	0.4315	0.5307	0.056*	
C53	0.0180 (3)	0.6550 (3)	0.91963 (16)	0.0654 (9)	
H53A	0.0243	0.5777	0.9532	0.078*	
H53B	−0.0096	0.7099	0.9506	0.078*	
C54	0.1393 (3)	0.6531 (3)	0.88536 (16)	0.0584 (8)	
H54A	0.1344	0.7290	0.8500	0.070*	
H54B	0.1946	0.6355	0.9250	0.070*	
C55	0.3064 (2)	0.5230 (2)	0.83743 (17)	0.0543 (8)	
H55A	0.3427	0.4785	0.8866	0.065*	
H55B	0.3375	0.5873	0.8154	0.065*	
C56	0.3365 (2)	0.4479 (2)	0.78696 (18)	0.0548 (8)	
H56A	0.4234	0.4075	0.7850	0.066*	
H56B	0.2972	0.3893	0.8060	0.066*	
C57	0.3517 (2)	0.4597 (3)	0.6580 (2)	0.0624 (9)	
H57A	0.3403	0.3842	0.6715	0.075*	
H57B	0.4381	0.4461	0.6540	0.075*	
C58	0.2974 (3)	0.5320 (3)	0.58454 (19)	0.0611 (9)	
H58A	0.3043	0.6091	0.5737	0.073*	
H58B	0.3433	0.4969	0.5446	0.073*	
C59	0.183 (3)	0.834 (3)	1.007 (5)	0.148 (9)	0.50
H59A	0.2137	0.7681	0.9858	0.222*	0.50
H59B	0.1849	0.8064	1.0618	0.222*	0.50
H59C	0.2325	0.8827	0.9882	0.222*	0.50
C60	0.0578 (10)	0.9024 (10)	0.9840 (7)	0.129 (3)	0.50
H60A	0.0081	0.8529	1.0023	0.155*	0.50
H60B	0.0557	0.9294	0.9286	0.155*	0.50
C61	0.008 (3)	1.004 (2)	1.016 (2)	0.153 (7)	0.50
H61A	0.0668	1.0428	1.0119	0.183*	0.50
H61B	−0.0209	0.9830	1.0684	0.183*	0.50
C62	−0.0921 (9)	1.0770 (10)	0.9632 (7)	0.129 (3)	0.50
H62A	−0.1440	1.0330	0.9635	0.155*	0.50
H62B	−0.0604	1.1008	0.9114	0.155*	0.50
C63	−0.162 (3)	1.182 (3)	0.991 (5)	0.148 (9)	0.50
H63A	−0.2256	1.2331	0.9567	0.222*	0.50
H63B	−0.1083	1.2224	0.9925	0.222*	0.50
H62C	−0.1955	1.1572	1.0413	0.222*	0.50

### Atomic displacement parameters (Å^2^)

**Table:**

	*U*^11^	*U*^22^	*U*^33^	*U*^12^	*U*^13^	*U*^23^
Co1	0.02717 (16)	0.02066 (15)	0.02826 (17)	−0.00811 (12)	−0.00491 (12)	−0.00371 (12)
C1	0.0376 (14)	0.0356 (14)	0.0643 (18)	−0.0045 (11)	−0.0036 (13)	−0.0286 (14)
C2	0.0409 (15)	0.0491 (16)	0.0461 (16)	−0.0058 (12)	−0.0005 (12)	−0.0263 (13)
C3	0.0384 (14)	0.0371 (14)	0.0368 (14)	−0.0086 (11)	0.0005 (11)	−0.0115 (11)
C4	0.0273 (11)	0.0222 (11)	0.0303 (12)	−0.0082 (9)	−0.0020 (9)	−0.0042 (9)
C5	0.0268 (11)	0.0190 (10)	0.0338 (12)	−0.0076 (9)	−0.0040 (9)	−0.0066 (9)
C6	0.0386 (13)	0.0345 (13)	0.0342 (13)	−0.0121 (11)	−0.0044 (11)	−0.0068 (10)
C7	0.0415 (15)	0.0479 (16)	0.0292 (13)	−0.0159 (12)	−0.0030 (11)	−0.0022 (11)
C8	0.0327 (13)	0.0375 (14)	0.0386 (14)	−0.0140 (11)	−0.0045 (11)	0.0085 (11)
C9	0.0308 (13)	0.0206 (12)	0.0623 (18)	−0.0095 (10)	−0.0080 (12)	0.0049 (11)
C10	0.0308 (13)	0.0227 (12)	0.0690 (19)	−0.0075 (10)	−0.0039 (12)	−0.0116 (12)
C11	0.0269 (12)	0.0265 (12)	0.0542 (16)	−0.0062 (10)	−0.0062 (11)	−0.0144 (11)
C12	0.0246 (11)	0.0290 (12)	0.0348 (13)	−0.0073 (9)	−0.0033 (9)	−0.0097 (10)
C13	0.0229 (11)	0.0252 (11)	0.0314 (12)	−0.0080 (9)	−0.0050 (9)	−0.0051 (9)
C14	0.0226 (11)	0.0266 (12)	0.0467 (15)	−0.0100 (9)	−0.0053 (10)	0.0005 (10)
C15	0.0216 (11)	0.0247 (11)	0.0396 (13)	−0.0067 (9)	−0.0039 (9)	−0.0078 (10)
C16	0.0228 (11)	0.0231 (11)	0.0361 (13)	−0.0083 (9)	−0.0041 (9)	−0.0031 (9)
C17	0.0661 (19)	0.0477 (16)	0.0332 (14)	−0.0197 (14)	−0.0066 (13)	−0.0145 (12)
C18	0.0608 (18)	0.0502 (16)	0.0365 (15)	−0.0205 (14)	0.0084 (13)	−0.0178 (13)
C19	0.0411 (14)	0.0371 (14)	0.0423 (15)	−0.0147 (11)	0.0022 (11)	−0.0138 (11)
C20	0.0268 (12)	0.0210 (11)	0.0372 (13)	−0.0064 (9)	−0.0051 (10)	−0.0060 (9)
C21	0.0332 (12)	0.0199 (11)	0.0281 (12)	−0.0075 (9)	−0.0054 (10)	−0.0023 (9)
C22	0.0399 (14)	0.0279 (12)	0.0399 (14)	−0.0131 (11)	0.0016 (11)	−0.0049 (10)
C23	0.0414 (15)	0.0397 (15)	0.0590 (18)	−0.0194 (12)	0.0114 (13)	−0.0121 (13)
C24	0.0343 (14)	0.0372 (14)	0.073 (2)	−0.0174 (12)	−0.0005 (13)	−0.0154 (14)
C25	0.0391 (15)	0.0333 (14)	0.071 (2)	−0.0107 (11)	−0.0178 (14)	−0.0181 (13)
C26	0.0534 (17)	0.0378 (14)	0.0536 (17)	−0.0114 (13)	−0.0190 (14)	−0.0166 (13)
C27	0.0484 (15)	0.0274 (12)	0.0407 (14)	−0.0091 (11)	−0.0129 (12)	−0.0101 (11)
C28	0.0344 (12)	0.0189 (10)	0.0348 (13)	−0.0072 (9)	−0.0027 (10)	−0.0076 (9)
C29	0.0334 (12)	0.0170 (10)	0.0360 (13)	−0.0084 (9)	0.0003 (10)	−0.0059 (9)
C30	0.0339 (13)	0.0231 (12)	0.0575 (17)	−0.0095 (10)	−0.0056 (12)	−0.0114 (11)
C31	0.0364 (13)	0.0185 (11)	0.0371 (13)	−0.0068 (9)	−0.0061 (10)	−0.0083 (9)
C32	0.0308 (12)	0.0173 (10)	0.0412 (13)	−0.0053 (9)	−0.0045 (10)	−0.0079 (9)
C33	0.0300 (12)	0.0242 (12)	0.0399 (14)	−0.0119 (10)	−0.0074 (10)	0.0004 (10)
C34	0.0320 (13)	0.0334 (13)	0.0290 (13)	−0.0143 (10)	−0.0042 (10)	−0.0011 (10)
C35	0.0313 (13)	0.0361 (13)	0.0478 (15)	−0.0151 (11)	−0.0011 (11)	−0.0077 (11)
C36	0.0317 (13)	0.0285 (12)	0.0520 (16)	−0.0072 (10)	−0.0138 (11)	−0.0062 (11)
C37	0.0359 (13)	0.0260 (12)	0.0340 (13)	−0.0109 (10)	−0.0125 (10)	−0.0021 (10)
C38	0.0372 (13)	0.0340 (13)	0.0293 (13)	−0.0135 (11)	−0.0089 (11)	−0.0045 (10)
C39	0.0398 (14)	0.0360 (13)	0.0462 (15)	−0.0121 (11)	−0.0121 (11)	−0.0143 (11)
C40	0.0374 (13)	0.0269 (12)	0.0527 (16)	−0.0125 (10)	−0.0123 (12)	−0.0083 (11)
K1	0.0304 (3)	0.0288 (3)	0.0311 (3)	−0.0072 (2)	−0.0031 (2)	−0.0058 (2)
N1	0.0541 (14)	0.0572 (14)	0.0372 (12)	−0.0345 (12)	0.0118 (10)	−0.0195 (11)
N2	0.0510 (13)	0.0439 (13)	0.0420 (13)	−0.0194 (11)	0.0043 (10)	−0.0173 (10)
O1	0.0291 (9)	0.0360 (10)	0.0614 (12)	−0.0055 (7)	0.0031 (8)	−0.0134 (9)
O2	0.0578 (12)	0.0309 (9)	0.0412 (10)	−0.0145 (8)	−0.0106 (9)	0.0003 (8)
O3	0.0415 (10)	0.0334 (9)	0.0395 (10)	−0.0143 (8)	−0.0011 (8)	−0.0067 (7)
O4	0.0353 (9)	0.0410 (10)	0.0454 (10)	−0.0125 (8)	−0.0021 (8)	−0.0163 (8)
O5	0.0497 (11)	0.0594 (12)	0.0503 (11)	−0.0278 (10)	−0.0146 (9)	−0.0104 (9)
O6	0.0370 (10)	0.0307 (9)	0.0663 (12)	−0.0025 (8)	−0.0094 (9)	−0.0183 (9)
C41	0.081 (2)	0.062 (2)	0.074 (2)	−0.0425 (18)	0.0431 (19)	−0.0442 (18)
C42	0.0476 (17)	0.0327 (15)	0.095 (3)	−0.0077 (13)	0.0281 (17)	−0.0180 (16)
C43	0.0347 (15)	0.0352 (15)	0.094 (2)	0.0024 (12)	−0.0268 (16)	−0.0109 (15)
C44	0.0595 (18)	0.0335 (14)	0.0543 (18)	−0.0102 (13)	−0.0280 (15)	0.0046 (12)
C45	0.082 (2)	0.0545 (17)	0.0290 (14)	−0.0363 (16)	−0.0028 (14)	−0.0040 (12)
C46	0.088 (2)	0.0606 (19)	0.0351 (15)	−0.0396 (17)	0.0019 (15)	−0.0178 (14)
C47	0.0507 (16)	0.0560 (17)	0.0419 (15)	−0.0296 (14)	0.0136 (12)	−0.0181 (13)
C48	0.0436 (15)	0.0506 (16)	0.0384 (15)	−0.0226 (13)	0.0011 (12)	−0.0054 (12)
C49	0.0453 (16)	0.0315 (13)	0.0586 (18)	−0.0144 (12)	0.0001 (13)	−0.0062 (12)
C50	0.0397 (15)	0.0414 (15)	0.0662 (19)	−0.0168 (12)	0.0005 (13)	−0.0208 (14)
C51	0.0484 (16)	0.0406 (15)	0.0500 (16)	−0.0123 (12)	−0.0100 (13)	−0.0189 (12)
C52	0.0529 (17)	0.0426 (15)	0.0490 (16)	−0.0129 (13)	0.0004 (13)	−0.0231 (13)
C53	0.084 (2)	0.107 (3)	0.0357 (16)	−0.067 (2)	0.0149 (15)	−0.0278 (17)
C54	0.073 (2)	0.087 (2)	0.0383 (16)	−0.0528 (19)	−0.0003 (14)	−0.0197 (16)
C55	0.0478 (17)	0.0512 (17)	0.0626 (19)	−0.0215 (14)	−0.0312 (14)	0.0047 (14)
C56	0.0389 (15)	0.0308 (14)	0.089 (2)	−0.0071 (12)	−0.0277 (15)	−0.0038 (15)
C57	0.0299 (14)	0.064 (2)	0.109 (3)	−0.0060 (13)	0.0011 (16)	−0.059 (2)
C58	0.0556 (19)	0.076 (2)	0.073 (2)	−0.0348 (17)	0.0239 (17)	−0.0469 (19)
C59	0.184 (12)	0.192 (10)	0.113 (7)	−0.120 (11)	−0.002 (13)	−0.038 (10)
C60	0.092 (5)	0.113 (6)	0.187 (9)	−0.004 (4)	−0.045 (5)	−0.066 (6)
C61	0.074 (10)	0.096 (7)	0.26 (3)	−0.044 (7)	0.044 (12)	−0.011 (11)
C62	0.092 (5)	0.113 (6)	0.187 (9)	−0.004 (4)	−0.045 (5)	−0.066 (6)
C63	0.184 (12)	0.192 (10)	0.113 (7)	−0.120 (11)	−0.002 (13)	−0.038 (10)

### Geometric parameters (Å, º)

**Table:**

Co1—C34	2.067 (2)	K1—O2	2.7810 (17)
Co1—C5	2.078 (2)	K1—O6	2.7989 (18)
Co1—C38	2.083 (2)	K1—O1	2.8106 (17)
Co1—C33	2.089 (2)	K1—O3	2.8208 (17)
Co1—C37	2.091 (2)	K1—O4	2.8237 (17)
Co1—C4	2.092 (2)	K1—N1	3.048 (2)
Co1—C20	2.093 (2)	K1—N2	3.074 (2)
Co1—C21	2.129 (2)	N1—C53	1.463 (4)
C1—C2	1.374 (4)	N1—C41	1.467 (4)
C1—C11	1.399 (4)	N1—C47	1.471 (3)
C1—H1	0.9500	N2—C52	1.459 (3)
C2—C3	1.395 (3)	N2—C58	1.463 (4)
C2—H2	0.9500	N2—C46	1.467 (4)
C3—C12	1.391 (3)	O1—C42	1.422 (3)
C3—H3	0.9500	O1—C43	1.433 (3)
C4—C5	1.420 (3)	O2—C45	1.413 (3)
C4—C12	1.453 (3)	O2—C44	1.420 (3)
C4—H4	0.96 (2)	O3—C49	1.421 (3)
C5—C13	1.460 (3)	O3—C48	1.422 (3)
C5—H5	0.95 (2)	O4—C51	1.416 (3)
C6—C13	1.387 (3)	O4—C50	1.418 (3)
C6—C7	1.397 (3)	O5—C54	1.416 (3)
C6—H6	0.9500	O5—C55	1.427 (3)
C7—C8	1.374 (4)	O6—C57	1.420 (3)
C7—H7	0.9500	O6—C56	1.420 (3)
C8—C14	1.399 (3)	C41—C42	1.500 (5)
C8—H8	0.9500	C41—H41A	0.9900
C9—C10	1.340 (4)	C41—H41B	0.9900
C9—C14	1.432 (3)	C42—H42A	0.9900
C9—H9A	0.9500	C42—H42B	0.9900
C10—C11	1.439 (3)	C43—C44	1.477 (4)
C10—H10A	0.9500	C43—H43A	0.9900
C11—C15	1.423 (3)	C43—H43B	0.9900
C12—C15	1.426 (3)	C44—H44A	0.9900
C13—C16	1.423 (3)	C44—H44B	0.9900
C14—C16	1.422 (3)	C45—C46	1.483 (4)
C15—C16	1.435 (3)	C45—H45A	0.9900
C17—C18	1.375 (4)	C45—H45B	0.9900
C17—C27	1.405 (4)	C46—H46A	0.9900
C17—H17	0.9500	C46—H46B	0.9900
C18—C19	1.387 (4)	C47—C48	1.496 (4)
C18—H18	0.9500	C47—H47A	0.9900
C19—C28	1.398 (3)	C47—H47B	0.9900
C19—H19	0.9500	C48—H48A	0.9900
C20—C21	1.425 (3)	C48—H48B	0.9900
C20—C28	1.448 (3)	C49—C50	1.487 (4)
C20—H20	0.97 (2)	C49—H49A	0.9900
C21—C29	1.455 (3)	C49—H49B	0.9900
C21—H21	0.93 (2)	C50—H50A	0.9900
C22—C29	1.392 (3)	C50—H50B	0.9900
C22—C23	1.395 (3)	C51—C52	1.506 (4)
C22—H22	0.9500	C51—H51A	0.9900
C23—C24	1.370 (4)	C51—H51B	0.9900
C23—H23	0.9500	C52—H52A	0.9900
C24—C30	1.404 (4)	C52—H52B	0.9900
C24—H24	0.9500	C53—C54	1.509 (4)
C25—C26	1.344 (4)	C53—H53A	0.9900
C25—C30	1.435 (4)	C53—H53B	0.9900
C25—H25	0.9500	C54—H54A	0.9900
C26—C27	1.432 (4)	C54—H54B	0.9900
C26—H26	0.9500	C55—C56	1.480 (4)
C27—C31	1.421 (3)	C55—H55A	0.9900
C28—C31	1.427 (3)	C55—H55B	0.9900
C29—C32	1.422 (3)	C56—H56A	0.9900
C30—C32	1.418 (3)	C56—H56B	0.9900
C31—C32	1.440 (3)	C57—C58	1.492 (5)
C33—C34	1.383 (3)	C57—H57A	0.9900
C33—C40	1.524 (3)	C57—H57B	0.9900
C33—H33	0.98 (2)	C58—H58A	0.9900
C34—C35	1.519 (3)	C58—H58B	0.9900
C34—H34	0.90 (2)	C59—C60	1.50 (3)
C35—C36	1.530 (3)	C59—H59A	0.9800
C35—H35A	0.9900	C59—H59B	0.9800
C35—H35B	0.9900	C59—H59C	0.9800
C36—C37	1.522 (3)	C60—C61	1.504 (9)
C36—H36A	0.9900	C60—H60A	0.9900
C36—H36B	0.9900	C60—H60B	0.9900
C37—C38	1.391 (3)	C61—C62	1.507 (9)
C37—H37	0.95 (2)	C61—H61A	0.9900
C38—C39	1.511 (3)	C61—H61B	0.9900
C38—H38	0.94 (2)	C62—C63	1.51 (3)
C39—C40	1.536 (3)	C62—H62A	0.9900
C39—H39A	0.9900	C62—H62B	0.9900
C39—H39B	0.9900	C63—H63A	0.9800
C40—H40A	0.9900	C63—H63B	0.9800
C40—H40B	0.9900	C63—H62C	0.9800
K1—O5	2.7735 (18)		
			
C34—Co1—C5	104.80 (9)	O5—K1—O3	89.39 (5)
C34—Co1—C38	101.05 (10)	O2—K1—O3	121.84 (5)
C5—Co1—C38	132.69 (9)	O6—K1—O3	123.64 (5)
C34—Co1—C33	38.88 (9)	O1—K1—O3	95.76 (5)
C5—Co1—C33	93.32 (9)	O5—K1—O4	114.96 (5)
C38—Co1—C33	82.73 (9)	O2—K1—O4	94.50 (5)
C34—Co1—C37	83.55 (10)	O6—K1—O4	88.43 (5)
C5—Co1—C37	170.29 (9)	O1—K1—O4	131.57 (5)
C38—Co1—C37	38.94 (9)	O3—K1—O4	60.80 (5)
C33—Co1—C37	90.20 (9)	O5—K1—N1	59.07 (6)
C34—Co1—C4	130.49 (9)	O2—K1—N1	121.33 (6)
C5—Co1—C4	39.81 (8)	O6—K1—N1	119.57 (6)
C38—Co1—C4	93.94 (9)	O1—K1—N1	60.79 (6)
C33—Co1—C4	98.53 (9)	O3—K1—N1	60.29 (5)
C37—Co1—C4	130.65 (9)	O4—K1—N1	120.75 (5)
C34—Co1—C20	90.25 (9)	O5—K1—N2	120.56 (6)
C5—Co1—C20	98.15 (9)	O2—K1—N2	59.08 (6)
C38—Co1—C20	120.80 (9)	O6—K1—N2	60.08 (6)
C33—Co1—C20	128.96 (9)	O1—K1—N2	119.61 (6)
C37—Co1—C20	86.60 (9)	O3—K1—N2	119.65 (5)
C4—Co1—C20	121.38 (9)	O4—K1—N2	59.13 (5)
C34—Co1—C21	129.33 (9)	N1—K1—N2	179.58 (7)
C5—Co1—C21	91.27 (9)	C53—N1—C41	110.7 (2)
C38—Co1—C21	102.03 (9)	C53—N1—C47	110.0 (2)
C33—Co1—C21	168.19 (9)	C41—N1—C47	110.7 (2)
C37—Co1—C21	87.03 (9)	C53—N1—K1	110.29 (17)
C4—Co1—C21	91.96 (9)	C41—N1—K1	106.74 (16)
C20—Co1—C21	39.43 (9)	C47—N1—K1	108.34 (14)
C2—C1—C11	120.4 (2)	C52—N2—C58	111.4 (2)
C2—C1—H1	119.8	C52—N2—C46	109.4 (2)
C11—C1—H1	119.8	C58—N2—C46	111.5 (2)
C1—C2—C3	120.4 (2)	C52—N2—K1	109.78 (15)
C1—C2—H2	119.8	C58—N2—K1	105.63 (15)
C3—C2—H2	119.8	C46—N2—K1	109.06 (16)
C12—C3—C2	121.7 (2)	C42—O1—C43	111.1 (2)
C12—C3—H3	119.2	C42—O1—K1	115.73 (15)
C2—C3—H3	119.2	C43—O1—K1	109.12 (15)
C5—C4—C12	119.7 (2)	C45—O2—C44	112.7 (2)
C5—C4—Co1	69.57 (12)	C45—O2—K1	116.06 (15)
C12—C4—Co1	122.28 (15)	C44—O2—K1	116.55 (15)
C5—C4—H4	117.6 (14)	C49—O3—C48	111.70 (19)
C12—C4—H4	115.8 (14)	C49—O3—K1	111.61 (14)
Co1—C4—H4	102.8 (14)	C48—O3—K1	114.58 (13)
C4—C5—C13	120.27 (19)	C51—O4—C50	111.35 (19)
C4—C5—Co1	70.62 (12)	C51—O4—K1	115.36 (14)
C13—C5—Co1	122.81 (15)	C50—O4—K1	113.36 (14)
C4—C5—H5	119.6 (13)	C54—O5—C55	113.6 (2)
C13—C5—H5	112.4 (13)	C54—O5—K1	113.55 (16)
Co1—C5—H5	104.0 (13)	C55—O5—K1	116.69 (16)
C13—C6—C7	121.0 (2)	C57—O6—C56	112.4 (2)
C13—C6—H6	119.5	C57—O6—K1	119.78 (15)
C7—C6—H6	119.5	C56—O6—K1	108.17 (16)
C8—C7—C6	120.4 (2)	N1—C41—C42	113.8 (2)
C8—C7—H7	119.8	N1—C41—H41A	108.8
C6—C7—H7	119.8	C42—C41—H41A	108.8
C7—C8—C14	121.0 (2)	N1—C41—H41B	108.8
C7—C8—H8	119.5	C42—C41—H41B	108.8
C14—C8—H8	119.5	H41A—C41—H41B	107.7
C10—C9—C14	121.7 (2)	O1—C42—C41	108.8 (2)
C10—C9—H9A	119.1	O1—C42—H42A	109.9
C14—C9—H9A	119.1	C41—C42—H42A	109.9
C9—C10—C11	121.9 (2)	O1—C42—H42B	109.9
C9—C10—H10A	119.1	C41—C42—H42B	109.9
C11—C10—H10A	119.1	H42A—C42—H42B	108.3
C1—C11—C15	119.6 (2)	O1—C43—C44	109.2 (2)
C1—C11—C10	122.2 (2)	O1—C43—H43A	109.8
C15—C11—C10	118.1 (2)	C44—C43—H43A	109.8
C3—C12—C15	118.1 (2)	O1—C43—H43B	109.8
C3—C12—C4	121.9 (2)	C44—C43—H43B	109.8
C15—C12—C4	119.9 (2)	H43A—C43—H43B	108.3
C6—C13—C16	118.9 (2)	O2—C44—C43	108.4 (2)
C6—C13—C5	121.6 (2)	O2—C44—H44A	110.0
C16—C13—C5	119.5 (2)	C43—C44—H44A	110.0
C8—C14—C16	119.0 (2)	O2—C44—H44B	110.0
C8—C14—C9	122.6 (2)	C43—C44—H44B	110.0
C16—C14—C9	118.5 (2)	H44A—C44—H44B	108.4
C11—C15—C12	119.7 (2)	O2—C45—C46	108.4 (2)
C11—C15—C16	119.9 (2)	O2—C45—H45A	110.0
C12—C15—C16	120.36 (19)	C46—C45—H45A	110.0
C14—C16—C13	119.8 (2)	O2—C45—H45B	110.0
C14—C16—C15	119.9 (2)	C46—C45—H45B	110.0
C13—C16—C15	120.34 (19)	H45A—C45—H45B	108.4
C18—C17—C27	120.1 (2)	N2—C46—C45	113.7 (2)
C18—C17—H17	120.0	N2—C46—H46A	108.8
C27—C17—H17	120.0	C45—C46—H46A	108.8
C17—C18—C19	120.9 (3)	N2—C46—H46B	108.8
C17—C18—H18	119.5	C45—C46—H46B	108.8
C19—C18—H18	119.5	H46A—C46—H46B	107.7
C18—C19—C28	121.6 (2)	N1—C47—C48	112.5 (2)
C18—C19—H19	119.2	N1—C47—H47A	109.1
C28—C19—H19	119.2	C48—C47—H47A	109.1
C21—C20—C28	119.6 (2)	N1—C47—H47B	109.1
C21—C20—Co1	71.65 (12)	C48—C47—H47B	109.1
C28—C20—Co1	118.60 (15)	H47A—C47—H47B	107.8
C21—C20—H20	119.8 (13)	O3—C48—C47	109.3 (2)
C28—C20—H20	114.3 (13)	O3—C48—H48A	109.8
Co1—C20—H20	104.4 (13)	C47—C48—H48A	109.8
C20—C21—C29	119.7 (2)	O3—C48—H48B	109.8
C20—C21—Co1	68.92 (12)	C47—C48—H48B	109.8
C29—C21—Co1	124.87 (15)	H48A—C48—H48B	108.3
C20—C21—H21	121.6 (14)	O3—C49—C50	109.0 (2)
C29—C21—H21	110.8 (14)	O3—C49—H49A	109.9
Co1—C21—H21	104.6 (14)	C50—C49—H49A	109.9
C29—C22—C23	121.1 (2)	O3—C49—H49B	109.9
C29—C22—H22	119.4	C50—C49—H49B	109.9
C23—C22—H22	119.4	H49A—C49—H49B	108.3
C24—C23—C22	120.9 (2)	O4—C50—C49	108.9 (2)
C24—C23—H23	119.6	O4—C50—H50A	109.9
C22—C23—H23	119.6	C49—C50—H50A	109.9
C23—C24—C30	120.3 (2)	O4—C50—H50B	109.9
C23—C24—H24	119.8	C49—C50—H50B	109.9
C30—C24—H24	119.8	H50A—C50—H50B	108.3
C26—C25—C30	121.8 (2)	O4—C51—C52	108.4 (2)
C26—C25—H25	119.1	O4—C51—H51A	110.0
C30—C25—H25	119.1	C52—C51—H51A	110.0
C25—C26—C27	121.7 (2)	O4—C51—H51B	110.0
C25—C26—H26	119.2	C52—C51—H51B	110.0
C27—C26—H26	119.2	H51A—C51—H51B	108.4
C17—C27—C31	119.4 (2)	N2—C52—C51	113.0 (2)
C17—C27—C26	122.2 (2)	N2—C52—H52A	109.0
C31—C27—C26	118.3 (2)	C51—C52—H52A	109.0
C19—C28—C31	117.8 (2)	N2—C52—H52B	109.0
C19—C28—C20	121.9 (2)	C51—C52—H52B	109.0
C31—C28—C20	120.3 (2)	H52A—C52—H52B	107.8
C22—C29—C32	118.3 (2)	N1—C53—C54	113.1 (2)
C22—C29—C21	121.6 (2)	N1—C53—H53A	109.0
C32—C29—C21	120.0 (2)	C54—C53—H53A	109.0
C24—C30—C32	119.2 (2)	N1—C53—H53B	109.0
C24—C30—C25	122.4 (2)	C54—C53—H53B	109.0
C32—C30—C25	118.4 (2)	H53A—C53—H53B	107.8
C27—C31—C28	120.1 (2)	O5—C54—C53	107.3 (2)
C27—C31—C32	120.0 (2)	O5—C54—H54A	110.2
C28—C31—C32	119.9 (2)	C53—C54—H54A	110.2
C30—C32—C29	120.2 (2)	O5—C54—H54B	110.2
C30—C32—C31	119.7 (2)	C53—C54—H54B	110.2
C29—C32—C31	120.2 (2)	H54A—C54—H54B	108.5
C34—C33—C40	125.4 (2)	O5—C55—C56	108.2 (2)
C34—C33—Co1	69.70 (13)	O5—C55—H55A	110.1
C40—C33—Co1	113.60 (16)	C56—C55—H55A	110.1
C34—C33—H33	116.7 (13)	O5—C55—H55B	110.1
C40—C33—H33	113.8 (13)	C56—C55—H55B	110.1
Co1—C33—H33	107.1 (12)	H55A—C55—H55B	108.4
C33—C34—C35	122.8 (2)	O6—C56—C55	108.7 (2)
C33—C34—Co1	71.42 (14)	O6—C56—H56A	109.9
C35—C34—Co1	110.95 (15)	C55—C56—H56A	109.9
C33—C34—H34	118.5 (15)	O6—C56—H56B	109.9
C35—C34—H34	114.8 (15)	C55—C56—H56B	109.9
Co1—C34—H34	107.1 (15)	H56A—C56—H56B	108.3
C34—C35—C36	111.8 (2)	O6—C57—C58	109.4 (2)
C34—C35—H35A	109.3	O6—C57—H57A	109.8
C36—C35—H35A	109.3	C58—C57—H57A	109.8
C34—C35—H35B	109.3	O6—C57—H57B	109.8
C36—C35—H35B	109.3	C58—C57—H57B	109.8
H35A—C35—H35B	107.9	H57A—C57—H57B	108.2
C37—C36—C35	112.64 (19)	N2—C58—C57	114.1 (3)
C37—C36—H36A	109.1	N2—C58—H58A	108.7
C35—C36—H36A	109.1	C57—C58—H58A	108.7
C37—C36—H36B	109.1	N2—C58—H58B	108.7
C35—C36—H36B	109.1	C57—C58—H58B	108.7
H36A—C36—H36B	107.8	H58A—C58—H58B	107.6
C38—C37—C36	125.5 (2)	C60—C59—H59A	109.5
C38—C37—Co1	70.23 (13)	C60—C59—H59B	109.5
C36—C37—Co1	112.29 (16)	H59A—C59—H59B	109.5
C38—C37—H37	116.2 (14)	C60—C59—H59C	109.5
C36—C37—H37	114.3 (14)	H59A—C59—H59C	109.5
Co1—C37—H37	107.3 (14)	H59B—C59—H59C	109.5
C37—C38—C39	122.9 (2)	C59—C60—C61	111 (2)
C37—C38—Co1	70.83 (13)	C59—C60—H60A	109.5
C39—C38—Co1	111.38 (16)	C61—C60—H60A	109.5
C37—C38—H38	117.9 (15)	C59—C60—H60B	109.5
C39—C38—H38	114.2 (15)	C61—C60—H60B	109.5
Co1—C38—H38	109.7 (15)	H60A—C60—H60B	108.1
C38—C39—C40	112.1 (2)	C60—C61—C62	99.0 (10)
C38—C39—H39A	109.2	C60—C61—H61A	112.0
C40—C39—H39A	109.2	C62—C61—H61A	112.0
C38—C39—H39B	109.2	C60—C61—H61B	112.0
C40—C39—H39B	109.2	C62—C61—H61B	112.0
H39A—C39—H39B	107.9	H61A—C61—H61B	109.6
C33—C40—C39	111.38 (19)	C61—C62—C63	107 (3)
C33—C40—H40A	109.4	C61—C62—H62A	110.3
C39—C40—H40A	109.4	C63—C62—H62A	110.3
C33—C40—H40B	109.4	C61—C62—H62B	110.3
C39—C40—H40B	109.4	C63—C62—H62B	110.3
H40A—C40—H40B	108.0	H62A—C62—H62B	108.6
O5—K1—O2	145.58 (6)	C62—C63—H63A	109.5
O5—K1—O6	60.69 (6)	C62—C63—H63B	109.5
O2—K1—O6	105.31 (6)	H63A—C63—H63B	109.5
O5—K1—O1	105.64 (6)	C62—C63—H62C	109.5
O2—K1—O1	60.77 (5)	H63A—C63—H62C	109.5
O6—K1—O1	135.93 (5)	H63B—C63—H62C	109.5
			
C11—C1—C2—C3	−0.4 (4)	C34—Co1—C38—C37	64.93 (15)
C1—C2—C3—C12	−2.1 (4)	C5—Co1—C38—C37	−172.78 (14)
C34—Co1—C4—C5	−60.02 (17)	C33—Co1—C38—C37	99.40 (15)
C38—Co1—C4—C5	−168.16 (13)	C4—Co1—C38—C37	−162.49 (15)
C33—Co1—C4—C5	−84.93 (14)	C20—Co1—C38—C37	−32.05 (18)
C37—Co1—C4—C5	177.41 (13)	C21—Co1—C38—C37	−69.64 (15)
C20—Co1—C4—C5	61.81 (15)	C34—Co1—C38—C39	−53.84 (19)
C21—Co1—C4—C5	89.64 (13)	C5—Co1—C38—C39	68.4 (2)
C34—Co1—C4—C12	52.9 (2)	C33—Co1—C38—C39	−19.38 (18)
C5—Co1—C4—C12	112.9 (2)	C37—Co1—C38—C39	−118.8 (2)
C38—Co1—C4—C12	−55.25 (19)	C4—Co1—C38—C39	78.74 (18)
C33—Co1—C4—C12	28.0 (2)	C20—Co1—C38—C39	−150.82 (16)
C37—Co1—C4—C12	−69.7 (2)	C21—Co1—C38—C39	171.59 (17)
C20—Co1—C4—C12	174.72 (17)	C37—C38—C39—C40	−48.1 (3)
C21—Co1—C4—C12	−157.46 (19)	Co1—C38—C39—C40	32.3 (2)
C12—C4—C5—C13	1.0 (3)	C34—C33—C40—C39	94.8 (3)
Co1—C4—C5—C13	117.4 (2)	Co1—C33—C40—C39	13.7 (2)
C12—C4—C5—Co1	−116.3 (2)	C38—C39—C40—C33	−29.8 (3)
C34—Co1—C5—C4	137.05 (13)	O5—K1—N1—C53	2.07 (17)
C38—Co1—C5—C4	16.17 (18)	O2—K1—N1—C53	−137.48 (17)
C33—Co1—C5—C4	99.35 (14)	O6—K1—N1—C53	−2.98 (19)
C37—Co1—C5—C4	−11.7 (6)	O1—K1—N1—C53	−131.97 (19)
C20—Co1—C5—C4	−130.52 (13)	O3—K1—N1—C53	111.19 (19)
C21—Co1—C5—C4	−91.53 (13)	O4—K1—N1—C53	104.54 (18)
C34—Co1—C5—C13	22.9 (2)	N2—K1—N1—C53	30 (8)
C38—Co1—C5—C13	−98.0 (2)	O5—K1—N1—C41	122.41 (19)
C33—Co1—C5—C13	−14.79 (19)	O2—K1—N1—C41	−17.15 (19)
C37—Co1—C5—C13	−125.9 (5)	O6—K1—N1—C41	117.35 (18)
C4—Co1—C5—C13	−114.1 (2)	O1—K1—N1—C41	−11.63 (17)
C20—Co1—C5—C13	115.34 (19)	O3—K1—N1—C41	−128.47 (19)
C21—Co1—C5—C13	154.33 (19)	O4—K1—N1—C41	−135.13 (17)
C13—C6—C7—C8	−0.4 (4)	N2—K1—N1—C41	150 (8)
C6—C7—C8—C14	−0.2 (4)	O5—K1—N1—C47	−118.38 (18)
C14—C9—C10—C11	0.6 (4)	O2—K1—N1—C47	102.07 (17)
C2—C1—C11—C15	1.1 (4)	O6—K1—N1—C47	−123.43 (17)
C2—C1—C11—C10	−176.9 (2)	O1—K1—N1—C47	107.59 (18)
C9—C10—C11—C1	176.4 (2)	O3—K1—N1—C47	−9.25 (16)
C9—C10—C11—C15	−1.7 (3)	O4—K1—N1—C47	−15.91 (19)
C2—C3—C12—C15	3.7 (3)	N2—K1—N1—C47	−90 (8)
C2—C3—C12—C4	180.0 (2)	O5—K1—N2—C52	97.28 (17)
C5—C4—C12—C3	−176.1 (2)	O2—K1—N2—C52	−123.00 (18)
Co1—C4—C12—C3	100.5 (2)	O6—K1—N2—C52	102.57 (17)
C5—C4—C12—C15	0.1 (3)	O1—K1—N2—C52	−128.64 (16)
Co1—C4—C12—C15	−83.3 (2)	O3—K1—N2—C52	−11.49 (18)
C7—C6—C13—C16	0.6 (3)	O4—K1—N2—C52	−5.32 (15)
C7—C6—C13—C5	−177.5 (2)	N1—K1—N2—C52	69 (8)
C4—C5—C13—C6	177.1 (2)	O5—K1—N2—C58	−22.92 (19)
Co1—C5—C13—C6	−97.5 (2)	O2—K1—N2—C58	116.80 (19)
C4—C5—C13—C16	−0.9 (3)	O6—K1—N2—C58	−17.62 (17)
Co1—C5—C13—C16	84.5 (2)	O1—K1—N2—C58	111.16 (18)
C7—C8—C14—C16	0.6 (3)	O3—K1—N2—C58	−131.69 (17)
C7—C8—C14—C9	−179.3 (2)	O4—K1—N2—C58	−125.51 (19)
C10—C9—C14—C8	−178.7 (2)	N1—K1—N2—C58	−51 (8)
C10—C9—C14—C16	1.4 (3)	O5—K1—N2—C46	−142.90 (16)
C1—C11—C15—C12	0.7 (3)	O2—K1—N2—C46	−3.18 (16)
C10—C11—C15—C12	178.7 (2)	O6—K1—N2—C46	−137.61 (18)
C1—C11—C15—C16	−177.3 (2)	O1—K1—N2—C46	−8.82 (18)
C10—C11—C15—C16	0.7 (3)	O3—K1—N2—C46	108.33 (17)
C3—C12—C15—C11	−3.0 (3)	O4—K1—N2—C46	114.50 (18)
C4—C12—C15—C11	−179.3 (2)	N1—K1—N2—C46	−171 (71)
C3—C12—C15—C16	175.0 (2)	O5—K1—O1—C42	−62.1 (2)
C4—C12—C15—C16	−1.3 (3)	O2—K1—O1—C42	152.4 (2)
C8—C14—C16—C13	−0.4 (3)	O6—K1—O1—C42	−125.86 (19)
C9—C14—C16—C13	179.5 (2)	O3—K1—O1—C42	28.9 (2)
C8—C14—C16—C15	177.9 (2)	O4—K1—O1—C42	84.5 (2)
C9—C14—C16—C15	−2.2 (3)	N1—K1—O1—C42	−22.24 (19)
C6—C13—C16—C14	−0.2 (3)	N2—K1—O1—C42	157.91 (19)
C5—C13—C16—C14	177.91 (19)	O5—K1—O1—C43	171.77 (16)
C6—C13—C16—C15	−178.4 (2)	O2—K1—O1—C43	26.19 (16)
C5—C13—C16—C15	−0.4 (3)	O6—K1—O1—C43	107.97 (17)
C11—C15—C16—C14	1.2 (3)	O3—K1—O1—C43	−97.25 (16)
C12—C15—C16—C14	−176.79 (19)	O4—K1—O1—C43	−41.72 (18)
C11—C15—C16—C13	179.5 (2)	N1—K1—O1—C43	−148.41 (17)
C12—C15—C16—C13	1.5 (3)	N2—K1—O1—C43	31.74 (17)
C27—C17—C18—C19	1.4 (4)	O5—K1—O2—C45	69.85 (19)
C17—C18—C19—C28	0.2 (4)	O6—K1—O2—C45	9.73 (17)
C34—Co1—C20—C21	−172.99 (14)	O1—K1—O2—C45	144.18 (17)
C5—Co1—C20—C21	82.02 (14)	O3—K1—O2—C45	−138.06 (16)
C38—Co1—C20—C21	−69.95 (16)	O4—K1—O2—C45	−79.87 (16)
C33—Co1—C20—C21	−176.96 (13)	N1—K1—O2—C45	149.70 (16)
C37—Co1—C20—C21	−89.46 (14)	N2—K1—O2—C45	−30.19 (16)
C4—Co1—C20—C21	47.26 (16)	O5—K1—O2—C44	−66.6 (2)
C34—Co1—C20—C28	72.67 (19)	O6—K1—O2—C44	−126.68 (17)
C5—Co1—C20—C28	−32.32 (19)	O1—K1—O2—C44	7.77 (16)
C38—Co1—C20—C28	175.71 (17)	O3—K1—O2—C44	85.53 (17)
C33—Co1—C20—C28	68.7 (2)	O4—K1—O2—C44	143.72 (17)
C37—Co1—C20—C28	156.20 (19)	N1—K1—O2—C44	13.29 (19)
C4—Co1—C20—C28	−67.1 (2)	N2—K1—O2—C44	−166.60 (19)
C21—Co1—C20—C28	−114.3 (2)	O5—K1—O3—C49	−98.89 (16)
C28—C20—C21—C29	−6.0 (3)	O2—K1—O3—C49	96.46 (16)
Co1—C20—C21—C29	−119.04 (19)	O6—K1—O3—C49	−45.41 (17)
C28—C20—C21—Co1	113.05 (19)	O1—K1—O3—C49	155.45 (15)
C34—Co1—C21—C20	9.08 (18)	O4—K1—O3—C49	20.42 (15)
C5—Co1—C21—C20	−101.32 (14)	N1—K1—O3—C49	−153.03 (17)
C38—Co1—C21—C20	124.41 (14)	N2—K1—O3—C49	26.49 (17)
C33—Co1—C21—C20	11.6 (5)	O5—K1—O3—C48	29.32 (15)
C37—Co1—C21—C20	88.25 (14)	O2—K1—O3—C48	−135.34 (15)
C4—Co1—C21—C20	−141.14 (14)	O6—K1—O3—C48	82.80 (16)
C34—Co1—C21—C29	121.4 (2)	O1—K1—O3—C48	−76.35 (15)
C5—Co1—C21—C29	11.0 (2)	O4—K1—O3—C48	148.62 (16)
C38—Co1—C21—C29	−123.3 (2)	N1—K1—O3—C48	−24.83 (15)
C33—Co1—C21—C29	123.9 (4)	N2—K1—O3—C48	154.69 (15)
C37—Co1—C21—C29	−159.5 (2)	O5—K1—O4—C51	−140.78 (15)
C4—Co1—C21—C29	−28.8 (2)	O2—K1—O4—C51	20.90 (15)
C20—Co1—C21—C29	112.3 (2)	O6—K1—O4—C51	−84.34 (15)
C29—C22—C23—C24	1.4 (4)	O1—K1—O4—C51	75.10 (16)
C22—C23—C24—C30	−0.6 (4)	O3—K1—O4—C51	145.11 (16)
C30—C25—C26—C27	−1.3 (4)	N1—K1—O4—C51	151.73 (15)
C18—C17—C27—C31	−1.3 (4)	N2—K1—O4—C51	−28.74 (15)
C18—C17—C27—C26	177.0 (2)	O5—K1—O4—C50	89.16 (16)
C25—C26—C27—C17	−175.4 (2)	O2—K1—O4—C50	−109.16 (16)
C25—C26—C27—C31	3.0 (4)	O6—K1—O4—C50	145.60 (16)
C18—C19—C28—C31	−1.7 (3)	O1—K1—O4—C50	−54.96 (18)
C18—C19—C28—C20	179.8 (2)	O3—K1—O4—C50	15.05 (15)
C21—C20—C28—C19	−179.5 (2)	N1—K1—O4—C50	21.67 (18)
Co1—C20—C28—C19	−95.4 (2)	N2—K1—O4—C50	−158.80 (17)
C21—C20—C28—C31	2.1 (3)	O2—K1—O5—C54	65.8 (2)
Co1—C20—C28—C31	86.2 (2)	O6—K1—O5—C54	139.32 (17)
C23—C22—C29—C32	−0.6 (3)	O1—K1—O5—C54	5.02 (17)
C23—C22—C29—C21	177.5 (2)	O3—K1—O5—C54	−90.79 (16)
C20—C21—C29—C22	−172.8 (2)	O4—K1—O5—C54	−147.89 (16)
Co1—C21—C29—C22	103.4 (2)	N1—K1—O5—C54	−35.64 (16)
C20—C21—C29—C32	5.3 (3)	N2—K1—O5—C54	144.59 (16)
Co1—C21—C29—C32	−78.6 (2)	O2—K1—O5—C55	−69.2 (2)
C23—C24—C30—C32	−0.9 (4)	O6—K1—O5—C55	4.30 (16)
C23—C24—C30—C25	178.0 (2)	O1—K1—O5—C55	−129.99 (17)
C26—C25—C30—C24	178.7 (2)	O3—K1—O5—C55	134.19 (17)
C26—C25—C30—C32	−2.4 (4)	O4—K1—O5—C55	77.09 (17)
C17—C27—C31—C28	−0.3 (3)	N1—K1—O5—C55	−170.66 (19)
C26—C27—C31—C28	−178.7 (2)	N2—K1—O5—C55	9.57 (19)
C17—C27—C31—C32	177.4 (2)	O5—K1—O6—C57	160.2 (2)
C26—C27—C31—C32	−1.0 (3)	O2—K1—O6—C57	−54.1 (2)
C19—C28—C31—C27	1.8 (3)	O1—K1—O6—C57	−117.6 (2)
C20—C28—C31—C27	−179.7 (2)	O3—K1—O6—C57	93.0 (2)
C19—C28—C31—C32	−175.9 (2)	O4—K1—O6—C57	40.2 (2)
C20—C28—C31—C32	2.6 (3)	N1—K1—O6—C57	165.1 (2)
C24—C30—C32—C29	1.6 (3)	N2—K1—O6—C57	−14.6 (2)
C25—C30—C32—C29	−177.3 (2)	O5—K1—O6—C56	29.56 (14)
C24—C30—C32—C31	−176.8 (2)	O2—K1—O6—C56	175.36 (14)
C25—C30—C32—C31	4.3 (3)	O1—K1—O6—C56	111.79 (15)
C22—C29—C32—C30	−0.9 (3)	O3—K1—O6—C56	−37.59 (16)
C21—C29—C32—C30	−179.02 (19)	O4—K1—O6—C56	−90.40 (15)
C22—C29—C32—C31	177.51 (19)	N1—K1—O6—C56	34.53 (16)
C21—C29—C32—C31	−0.6 (3)	N2—K1—O6—C56	−145.21 (16)
C27—C31—C32—C30	−2.6 (3)	C53—N1—C41—C42	164.6 (2)
C28—C31—C32—C30	175.10 (19)	C47—N1—C41—C42	−73.2 (3)
C27—C31—C32—C29	179.0 (2)	K1—N1—C41—C42	44.5 (3)
C28—C31—C32—C29	−3.3 (3)	C43—O1—C42—C41	179.6 (2)
C5—Co1—C33—C34	109.61 (15)	K1—O1—C42—C41	54.4 (3)
C38—Co1—C33—C34	−117.76 (15)	N1—C41—C42—O1	−69.1 (3)
C37—Co1—C33—C34	−79.44 (15)	C42—O1—C43—C44	171.9 (2)
C4—Co1—C33—C34	149.32 (14)	K1—O1—C43—C44	−59.3 (2)
C20—Co1—C33—C34	6.34 (19)	C45—O2—C44—C43	−177.7 (2)
C21—Co1—C33—C34	−3.1 (5)	K1—O2—C44—C43	−39.9 (3)
C34—Co1—C33—C40	120.8 (2)	O1—C43—C44—O2	67.6 (3)
C5—Co1—C33—C40	−129.62 (17)	C44—O2—C45—C46	−160.0 (2)
C38—Co1—C33—C40	3.01 (17)	K1—O2—C45—C46	62.0 (2)
C37—Co1—C33—C40	41.32 (18)	C52—N2—C46—C45	154.5 (2)
C4—Co1—C33—C40	−89.91 (17)	C58—N2—C46—C45	−81.8 (3)
C20—Co1—C33—C40	127.11 (17)	K1—N2—C46—C45	34.4 (3)
C21—Co1—C33—C40	117.6 (4)	O2—C45—C46—N2	−64.5 (3)
C40—C33—C34—C35	−1.7 (4)	C53—N1—C47—C48	−79.0 (3)
Co1—C33—C34—C35	103.4 (2)	C41—N1—C47—C48	158.4 (2)
C40—C33—C34—Co1	−105.1 (2)	K1—N1—C47—C48	41.6 (3)
C5—Co1—C34—C33	−76.58 (15)	C49—O3—C48—C47	−173.9 (2)
C38—Co1—C34—C33	63.42 (15)	K1—O3—C48—C47	58.0 (2)
C37—Co1—C34—C33	98.38 (15)	N1—C47—C48—O3	−68.9 (3)
C4—Co1—C34—C33	−41.57 (19)	C48—O3—C49—C50	176.5 (2)
C20—Co1—C34—C33	−175.08 (15)	K1—O3—C49—C50	−53.7 (2)
C21—Co1—C34—C33	179.17 (14)	C51—O4—C50—C49	179.9 (2)
C5—Co1—C34—C35	164.53 (16)	K1—O4—C50—C49	−48.0 (2)
C38—Co1—C34—C35	−55.47 (18)	O3—C49—C50—O4	69.8 (3)
C33—Co1—C34—C35	−118.9 (2)	C50—O4—C51—C52	−168.2 (2)
C37—Co1—C34—C35	−20.52 (17)	K1—O4—C51—C52	60.8 (2)
C4—Co1—C34—C35	−160.47 (15)	C58—N2—C52—C51	153.5 (2)
C20—Co1—C34—C35	66.03 (18)	C46—N2—C52—C51	−82.7 (3)
C21—Co1—C34—C35	60.3 (2)	K1—N2—C52—C51	36.9 (3)
C33—C34—C35—C36	−48.3 (3)	O4—C51—C52—N2	−66.1 (3)
Co1—C34—C35—C36	32.6 (2)	C41—N1—C53—C54	−89.0 (3)
C34—C35—C36—C37	−29.0 (3)	C47—N1—C53—C54	148.4 (3)
C35—C36—C37—C38	93.0 (3)	K1—N1—C53—C54	29.0 (3)
C35—C36—C37—Co1	12.0 (2)	C55—O5—C54—C53	−156.5 (2)
C34—Co1—C37—C38	−116.53 (16)	K1—O5—C54—C53	67.0 (3)
C5—Co1—C37—C38	33.2 (6)	N1—C53—C54—O5	−63.6 (3)
C33—Co1—C37—C38	−78.14 (15)	C54—O5—C55—C56	−171.3 (2)
C4—Co1—C37—C38	23.31 (19)	K1—O5—C55—C56	−36.3 (3)
C20—Co1—C37—C38	152.83 (15)	C57—O6—C56—C55	162.8 (2)
C21—Co1—C37—C38	113.34 (15)	K1—O6—C56—C55	−62.7 (2)
C34—Co1—C37—C36	4.76 (17)	O5—C55—C56—O6	67.6 (3)
C5—Co1—C37—C36	154.5 (5)	C56—O6—C57—C58	174.1 (2)
C38—Co1—C37—C36	121.3 (2)	K1—O6—C57—C58	45.4 (3)
C33—Co1—C37—C36	43.15 (17)	C52—N2—C58—C57	−69.6 (3)
C4—Co1—C37—C36	144.60 (15)	C46—N2—C58—C57	167.9 (2)
C20—Co1—C37—C36	−85.88 (17)	K1—N2—C58—C57	49.5 (3)
C21—Co1—C37—C36	−125.37 (17)	O6—C57—C58—N2	−66.3 (3)
C36—C37—C38—C39	−0.2 (4)	C59—C60—C61—C62	160 (4)
Co1—C37—C38—C39	103.6 (2)	C60—C61—C62—C63	175 (3)
C36—C37—C38—Co1	−103.8 (2)		
